# Synthesis, thermal behaviors, and energetic properties of asymmetrically substituted tetrazine-based energetic materials

**DOI:** 10.3389/fchem.2022.978003

**Published:** 2022-10-03

**Authors:** Shenghui Wang, Xiang Chen, Yuankai Chen, Hai Nan, Yuanyuan Li, Haixia Ma

**Affiliations:** ^1^ School of Chemical Engineering, Xi’an Key Laboratory of Special Energy Materials, Northwest University, Xi’an, Shaanxi, China; ^2^ Xi’an Modern Chemistry Research Institute, Xi’an, China

**Keywords:** tetrazine, asymmetrically substituted, crystal structure, thermal behavior, detonation properties

## Abstract

1,2,4,5-tetrazine ring is a common structure for the construction of energy-containing compounds, and its high nitrogen content and large conjugation effect give it the advantage of a good balance between energy and mechanical stability as a high-nitrogen energy-containing material. However, most of the reported works about tetrazine energetic materials (EMs) are symmetrically substituted tetrazines due to their easy accessibility. A small number of reports show that asymmetrically substituted tetrazines also have good properties, such as high density and generation of enthalpy and energy. Herein, two asymmetrically substituted tetrazines and their five energetic salts were prepared and fully characterized by IR spectroscopy, NMR spectra, elemental analysis, and differential scanning calorimetry (DSC). The structure of the two compounds was further confirmed by single-crystal X-ray diffraction studies. The thermal behaviors and thermodynamic parameters were determined and calculated. In addition, the energetic properties and impact sensitivities of all the compounds were obtained to assess their application potential. The results show that compounds **2**–**4** and **7**–**9** show higher detonation velocities than TNT, and the hydrazinium salt **9** possesses the best detonation properties (*D* = 8,232 m s^−1^ and *p* = 23.6 GPa). Except for **4** and **3**, all the other compounds are insensitive, which may be applied as insensitive explosives. Noncovalent interaction analysis was further carried out, and the result shows that the strong and high proportion of hydrogen bonds may contribute to the low-impact sensitivity.

## Introduction

**FIGURE 1:**
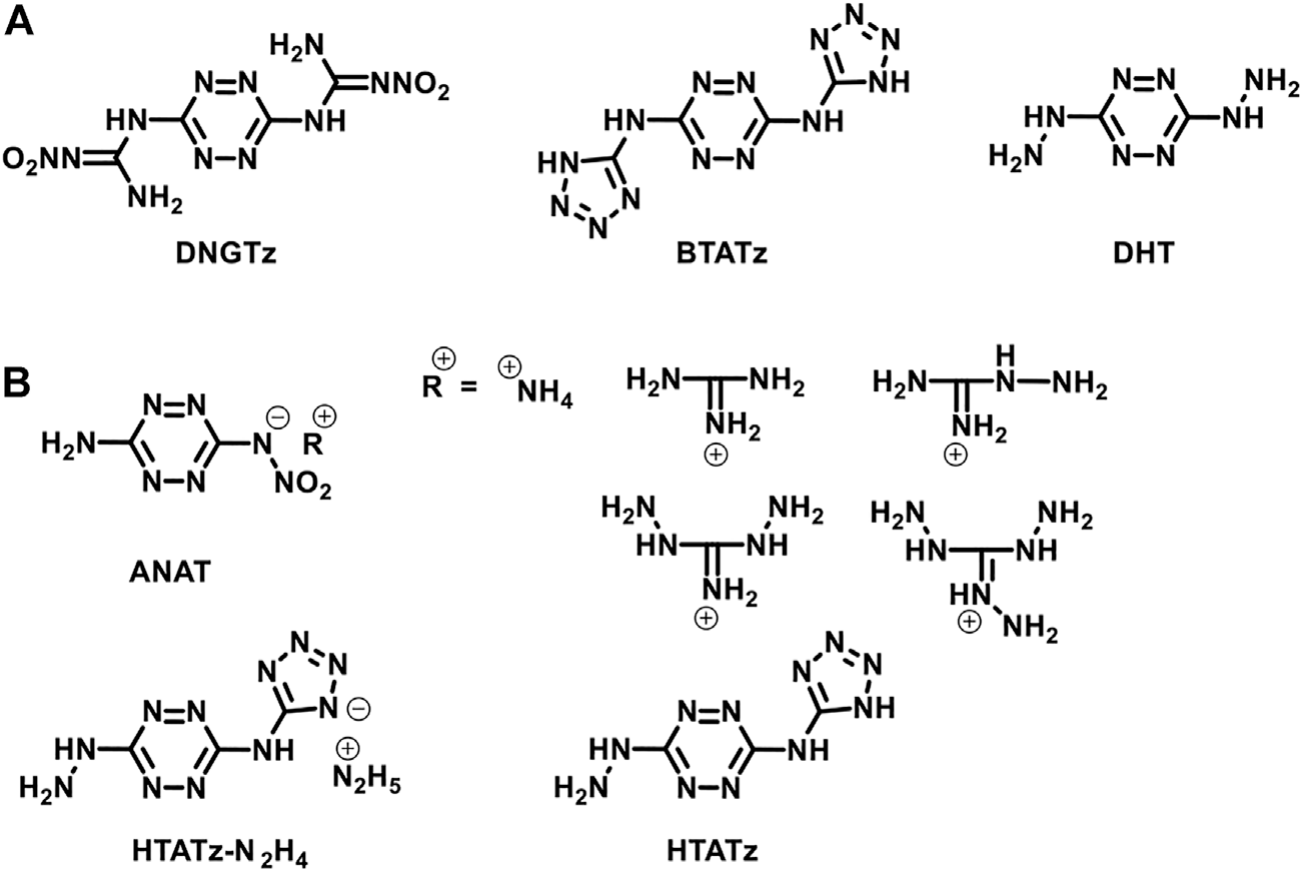
The structures of the representatives of symmetrically and asymmetrically substituted s-teterazine-based energetic materials.

Energetic materials have been developed for many years to satisfy the application of industrial and military. For example, trinitrotoluene (TNT), cyclotrimethylene trinitramine (RDX), cyclotetramethylene tetranitramine (HMX), and 1,3,5-triamino-2,4,6-trinitrobenzene (TATB) are represented as the three phases of energy-containing materials, respectively. The reason that hinders the development of energetic materials is the inherent contradiction between energy and safety (Gao et al., 2020; Gettings et al., 2020; Du et al., 2021; Muravyev et al., 2021; Zheng et al., 2021). The energy source of traditional nitramine explosives, such as CL-20 and HMX, is the strong redox reaction between the nitro group and the framework structure. Therefore, in order to obtain high energy, more nitro groups should be introduced into the skeleton. But the more these substituents are present, the poorer their stability. For the purpose of gaining high energy while keeping good stability, high nitrogen content energetic materials (HNCEMs) were developed in the past few decades (Lin et al., 2018; Tarchoun et al., 2020; Wu et al., 2020; Lei et al., 2021a; Feng et al., 2021; Herweyer et al., 2021; Tang et al., 2021). The common HNCEMs are pyrazole (Li et al., 2018; Zhang W. et al., 2020; Lei et al., 2021b; Lai et al., 2022), triazole (Manship et al., 2020; Wozniak et al., 2020; Yan et al., 2020), tetrazole (Gamekkanda et al., 2020; Yu et al., 2019; Jafari et al., 2018), and tetrazine (Chavez and Hiskey, 1999; Hiskey et al., 2001; Chavez et al., 2004; Gao et al., 2006; Sinditskii et al., 2012; Ma et al., 2021) heterocyclic compounds. Among them, tetrazine compounds have a high nitrogen content of 68.3%, and their skeleton shows good thermal stability and low mechanical sensitivity. Therefore, many tetrazine HNCEMs are reported, and the representatives are 6-bis-nitroguanyl-1,2,4,5-tetrazine (DNGTz) (Chavez et al., 2004), 3,6-bis(1H-1,2,3,4-tetrazol-5-ylamino)-1,2,4,5-tetrazine (BTATz) (Chavez and Hiskey, 1999), and 3,6-dihydrazino-1,2,4,5-tetrazine (DHT) (Hiskey et al., 2001) (Figure 1A). These materials show good balance between their detonation properties and mechanical sensitivity, which can be good candidates for many traditional explosives.

Similar to DNGTz, BTATz, and DHT, most of the representatives of tetrazine HNCEMs are symmetrically substituted tetrazines, which means that the substitutes on the tetrazine ring are the same, and the whole molecule shows good structural symmetry. Reports about the asymmetrically substituted tetrazines are seldom seen (Gao et al., 2006; Sinditskii et al., 2012). One of the most important reasons is that the synthesis of asymmetrically substituted tetrazines is more complex than the synthesis of symmetrically substituted tetrazines. The success of the reaction depends on whether the nucleophilic ability of the energetic group is strong enough. For example, Chaves et al. reported the preparation of asymmetrically substituted tetrazines based on 3,6-dichloro-1,2,4,5-tetrazine (Chavez and Hiskey, 1999). The results show that it is very difficult to replace the second chlorine atom and it needs several days at reflux to obtain a reasonable yield. However, energetic properties calculations revealed that the asymmetrically substituted tetrazines possess comparable detonation performances with SSTEs, which disclose their potential application prospects. For instance, Gao et al. synthesized several asymmetrically substituted tetrazines based on 3-amino-6-nitroamino-tetrazine (ANAT, Figure 1B) (Gao et al., 2006). These compounds exhibit detonation velocities higher than 8,000 m s^−1^, which is comparable to tetryl, PETN, TATB, and RDX. In addition, Sinditskii et al. reported the preparation of 3-hydrazino-6-(1H-1,2,3,4-tetrazol-5-ylimino)-1,2,4,5-tetrazine (HTATz, Figure 1B) (Sinditskii et al., 2012) and its hydrazine salt HTATz·N_2_H_4_. Both the compounds have high heat of formation greater than 600 kJ mol^−1^ (HTATz = 623 kJ mol^−1^, HTATz·N_2_H_4_ = 669 kJ mol^−1^) and high detonation velocities (HTATz = 8,100 m s^−1^, HTATz·N_2_H_4_ = 8,500 m s^−1^). We have reported our study on 6-((2H-tetrazol-5-yl)-amino)-1,2,4,5-tetrazin-3-one, which has a detonation velocity of 7,757 m s^−1^ and a detonation pressure of 25.7 GPa. (Zhang C. et al., 2020). All the abovementioned works reveal that it is meaningful to develop asymmetrically substituted tetrazines.

Given this background, for the purpose of continuing our study on asymmetrically substituted tetrazines, we report our recent work on synthesizing two asymmetrically substituted tetrazines and their salts. 3-hydrazinyl-6-(1H-pyrazol-1-yl)-1,2,4,5-tetrazine (**2**) and 3-hydroxyl-6-(1H-pyrazol-1-yl)-1,2,4,5-tetrazine (**5**) were chosen as the target compounds for the following two reasons. First, the nucleophilic ability of the hydrazine, pyrazole, and hydroxyl anion is strong enough to improve the efficiency of the nucleophilic substitution reaction. Second, the hydrazinyl and hydroxyl groups can further be protonated and deprotonated to obtain their energetic salts. Chavez et al. (2016) and Myers et al. (2016) have reported the syntheses of compounds **2** and **5**. In this work, we optimized the synthesis route of compound **5** and successfully obtained it under milder conditions. The nitrate (**3**), perchlorate (**4**), salts of (**2**) and the ammonium (**7**), hydroxylammonium (**8**), and hydrazinium (**9**) salts of (**5**) were also prepared and fully characterized. Single-crystal X-ray diffraction studies were performed on compounds (**7**) and (**8**). The thermal behaviors, thermodynamic parameters, energetic properties, and sensitivities were investigated. Noncovalent interaction analyses were also carried out to explain the mechanical stability.

## Experimental section

Caution should be exercised! Although no explosions were observed during the syntheses and handling of all the compounds, they must be synthesized only on a small scale, and mechanical actions including scratching or scraping should also be avoided. Eye protection, face shields, and leather gloves must be worn during the preparation.

### Materials and methods

All of the reagents were obtained from commercial sources and used without further purification. IR spectra were recorded using a Shimadzu IRAﬃnity-1S FTIR spectrophotometer (KBr pellets). Elemental analyses were carried out using a VarioEL III elemental analyzer (Elementar Co., Germany). 1H (500 MHz) and 13C (125 MHz) NMR spectra were performed on BRUKER AVANCE III HD. Chemical shifts were recorded relative to TMS. The thermal behaviors of all the compounds (Test sample mass is 0.2 mg) were studied on diﬀerential scanning calorimetry (DSC, Q2000, TA Co.) at a heating rate of 10°C min^-1^ in a N_2_ atmosphere with a flow rate of 50 ml·min^-1^ under ambient atmospheric pressure. To ensure the reproducibility and accuracy of the DSC analysis, each β value was repeatedly measured three times. Impact sensitivity test equipment used a vertical falling hammer instrument; test conditions: hammer mass (5.000 ± 0.005) kg, single test sample mass of 50 mg.

Single-crystal X-ray diﬀraction of compounds 4·H_2_O and 6 were determined using a Bruker D8 Venture diﬀractometer that was outfitted with a PHOTON-100 CMOS detector with highly oriented graphite crystal monochromated Mo-Kα radiation. Multi-scan spherical absorption correction was carried out. The structures of **4·H**_**2**_ **O** and **6** were solved by using ShelXT and refined by the full-matrix least-squares techniques based on *F* ^2^. Hydrogen atoms were refined using a riding model, while non-hydrogen atoms were refined anisotropically.

### Syntheses

**SCHEME 1:**
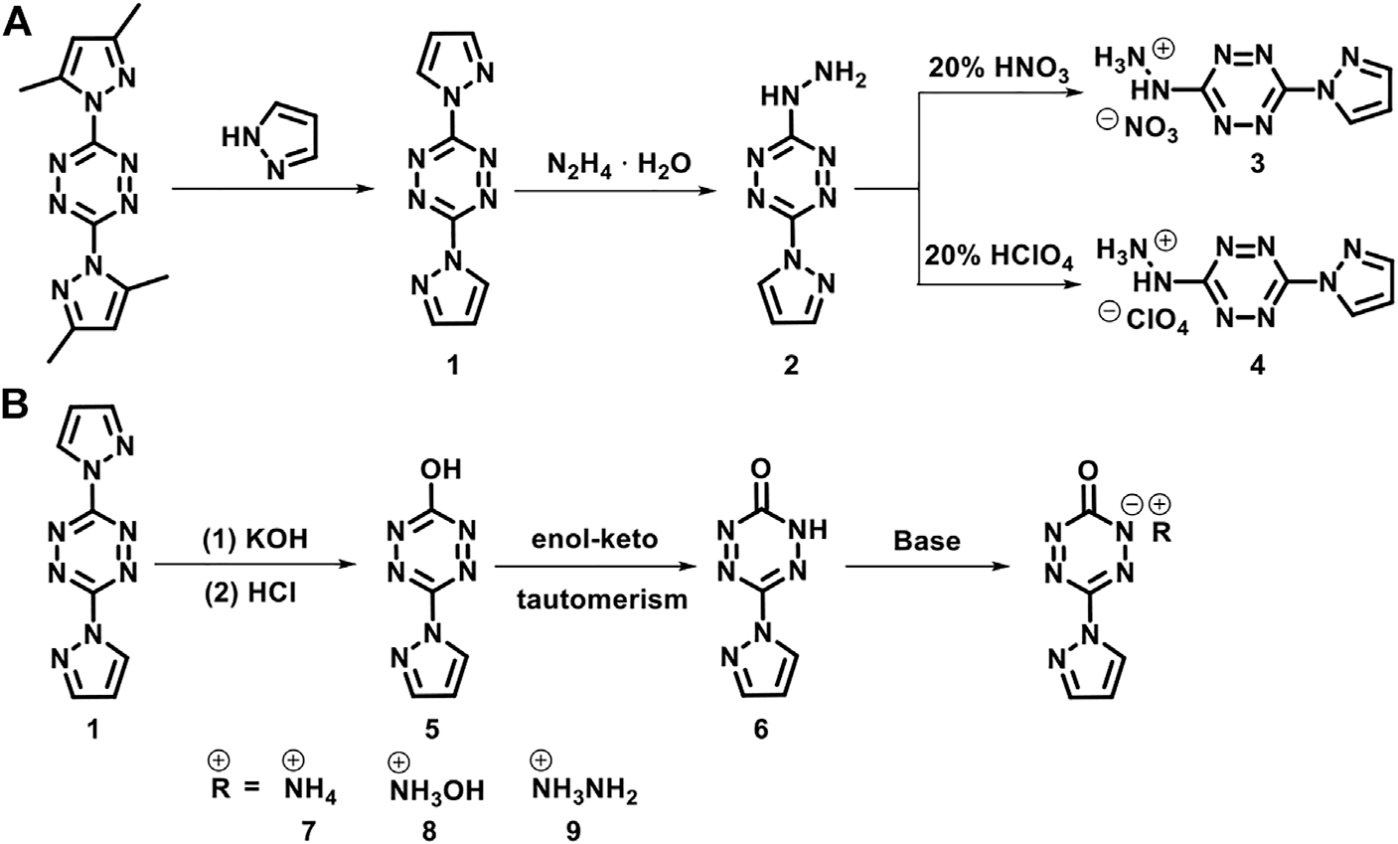
The synthetic routes of 3-hydrazinyl-6-(1H-pyrazol-1-yl)-1,2,4,5-tetrazine (2), 6-(1H-pyrazol-1-yl)-1,2,4,5-tetrazin-3(2H)-one (6) and their derivatives.

The synthetic routes are shown in Scheme 1. 3,6-bis(3,5-dimethyl-1H-pyrazol-1-yl)-1,2,4,5-tetrazine, 3,6-di(1H-pyrazol-1-yl)-1,2,4,5-tetrazine (**1**), and 3-hydrazinyl-6-(1H-pyrazol-1-yl)-1,2,4,5-tetrazine (**2**) were prepared according to the literature (Sinditskii et al., 2012).

#### 3-hydrazinyl-6-(1h-pyrazol-1-yl)-1,2,4,5- tetrazinium nitrate (**3**)

Compound **2** (1.78 g, 10 mmol) was added slowly to 20% nitric acid (15 ml) under stirring. An orange solid formed immediately. The mixture was kept at room temperature for 1 h. The precipitate was filtered, washed with isopropyl alcohol, and air-dried to yield compound **3** in 68% yield. Orange solid; ^1^H NMR (500 MHz, [D_6_]DMSO): *δ* = 2.39 (s, 1H), 6.71 (s, 1H), 7.16 (t, 3H), 7.98 (s, 1H), 8.66 (s, 1H) ppm; ^13^C NMR (125 MHz, [D_6_]DMSO): *δ* = 114.37, 134.45, 148.77, 161.89, 168.22 ppm; IR (KBr): *ṽ* = 3,128, 2,888, 2,365, 1,616, 1,559, 1,472, 1,396, 1,362, 1,319, 1,194, 1,035, 959, 940, 853, 825, 777, 603, and 541 cm^−1^; elemental analysis calcd (%) for C_5_H_7_N_9_O_3_ (241.17): C 24.90, H 2.93, N 52.27; found: C 24.58, H 2.62, N 51.91.

#### 3-hydrazinyl-6-(1H-pyrazol-1-yl)-1,2,4,5- tetrazinium perchlorate (**4**)

Compound **2** (1.78 g, 10 mmol) was added slowly to 20% perchloric acid (15 ml) under stirring. An orange solid formed immediately. The mixture was kept at room temperature for 1 h. Then, the precipitate was isolated by centrifugation, washed with isopropyl alcohol, and air-dried to yield compound **4** in 60% yield. Orange solid; ^1^H NMR (500 MHz, [D_6_]DMSO): *δ* = 6.76 (s, 1H), 8.04 (s, 1H), 8.75 (s, 1H), 11.19 (br, 3H) ppm; ^13^C NMR (125 MHz, [D_6_]DMSO): *δ* = 110.14, 130.08, 144.68, 157.80, 162.34 ppm; IR (KBr): *ṽ* = 3,243, 2,864, 2,691, 2,365, 1,611, 1,549, 1,501, 1,424, 1,343, 1,074, 1,040, 954, 781, 729, 685, 623, 565 cm^−1^; elemental analysis calcd (%) for C_5_H_7_N_8_O_4_Cl (278.61): C 21.55, H 2.53, N 40.22; found: C 21.38, H 2.40, N 40.05.

#### 6-(1H-pyrazol-1-yl)-1,2,4,5-tetrazin-3(2H)-one (**6**)

Compound **1** (2.14 g, 10 mmol) was slowly added to a solution of potassium hydroxide (0.12 mol L^−1^, 100 ml) in water under stirring. The suspension was stirred at room temperature for 0.5 h to obtain a clear solution. The insoluble impurities were removed by filtration. The filtrate was treated under reduced pressure to remove the solvent. A minimum amount of water was again added to dissolve the solid, and the solution was acidified to pH = 1 by using 5% hydrochloric acid. An orange-red precipitate formed. The precipitate was filtered, washed with water, and air-dried to obtain compound **6** in 71% yield. Orange-red solid; ^1^H NMR (500 MHz, [D_6_]DMSO): *δ* = 6.54 (s, 1H), 7.77 (s, 1H), 8.34 (s, 1H) ppm; ^13^C NMR (125 MHz, [D_6_]DMSO): *δ* = 107.65, 128.77, 141.56, 152.99, 166.21 ppm; IR (KBr): *ṽ* = 3,128, 3,056, 2,927, 2,826, 1746, 1,698, 1,587, 1,530, 1,453, 1,396, 1,309, 1,189, 1,107, 1,035, 992, 930, 868, 767, 666, 594 cm^−1^; elemental analysis calcd (%) for C_5_H_4_N_6_O (164.13): C 36.59, H 2.46, N 51.21; found: C 36.68, H 2.55, N 51.11.

### Synthesis of energetic salts **7**–**8**

Compound **6** (0.164 g, 1 mmol) was suspended in methanol (4 ml). Then, 25% aqueous ammonia (0.154 g, 1.1 mmol) and 50% aqueous hydroxylamine (0.073 g, 1.1 mmol) was slowly added under stirring. The solution was kept at room temperature for 2 h. Then, it was treated under reduced pressure to remove the solvent. A purplish-red solid formed and was air-dried to obtain compounds **7** and **8**.

#### Ammonium 6-(1H-pyrazol-1-yl)-1,2,4,5-tetrazin-3(2H)-one (**7**)

86% yield; ^1^H NMR (500 MHz, [D_6_]DMSO): *δ* = 6.54 (s, 1H), 7.27 (s, 4H), 7.76 (s, 1H), 8.33 (s, 1H) ppm; ^13^C NMR (125 MHz, [D_6_]DMSO): *δ* = 112.44, 133.60, 146.32, 157.93, and 171.19 ppm; IR (KBr): *ṽ* = 3,118, 2,979, 2,840, 1,544, 1,477, 1,420, 1,396, 1,300, 1,257, 1,194, 1,059, 1,031, 959, 853, 753, 695, 603, 575 cm^−1^; elemental analysis calcd (%) for C_5_H_7_N_7_O (181.16): C 33.15, H 3.89, N 54.12; found: C 32.81, H 3.53, N 53.78.

#### Hydroxylammonium 6-(1H-pyrazol-1-yl)-1,2,4,5-tetrazin-3(2H)-one (**8**)

88% yield; ^1^H NMR (500 MHz, [D_6_]DMSO): *δ* = 6.56 (s, 1H), 7.79 (s, 1H), 8.37 (s, 1H), 10.24 (br, 3H) ppm; ^13^C NMR (125 MHz, [D_6_]DMSO): *δ* = 112.62, 133.72, 146.64, 157.92, 171.06 ppm; IR (KBr): *ṽ* = 3,104, 2,927, 2,701, 1,515, 1,477, 1,396, 1,305, 1,194, 1,031, 949, 762, 695, 609, 575 cm^−1^; elemental analysis calcd (%) for C_5_H_7_N_7_O_2_ (197.16): C 30.46, H 3.58, N 49.73; found: C 30.31, H 3.63, N 49.58.

#### Hydrazinium 6-(1H-pyrazol-1-yl)-1,2,4,5-tetrazin-3(2H)-one (**9**)

50% hydrazine monohydride (0.110 g, 1.1 mmol) was slowly added under stirring to the suspension of compound **6** (0.164 g, 1 mmol) in methanol (4 ml). A purplish-red solid formed, and the mixture was stirred at room temperature for 2 h. The precipitate was filtered, washed with acetonitrile, and air-dried to obtain compound **9** in 85% yield. Purplish-red solid; ^1^H NMR (500 MHz, [D_6_]DMSO): *δ* = 6.52 (s, 1H), 7.76 (s, 5H), 8.09 (s, 1H), 8.31 (s, 1H) ppm; ^13^C NMR (125 MHz, [D_6_]DMSO): *δ* = 112.41, 133.63, 146.33, 157.90, 171.08 ppm; IR (KBr): *ṽ* = 3,325, 2,840, 2,725, 1,621, 1,554, 1,511, 1,477, 1,396, 1,343, 1,300, 1,199, 1,127, 1,093, 1,026, 959, 810, 753, 700, 614, 575 cm^−1^; elemental analysis calcd (%) for C_5_H_8_N_8_O (196.17): C 30.61, H 4.11, N 57.12; found: C 30.34, H 4.23, N 57.31.

## Results and discussion

### Crystallography

The single-crystals of **4·H**_**2**_ **O** and **6** were both grown from the filtrates. Crystallographic data are summarized in Supplementary Table S1.

**FIGURE 2:**
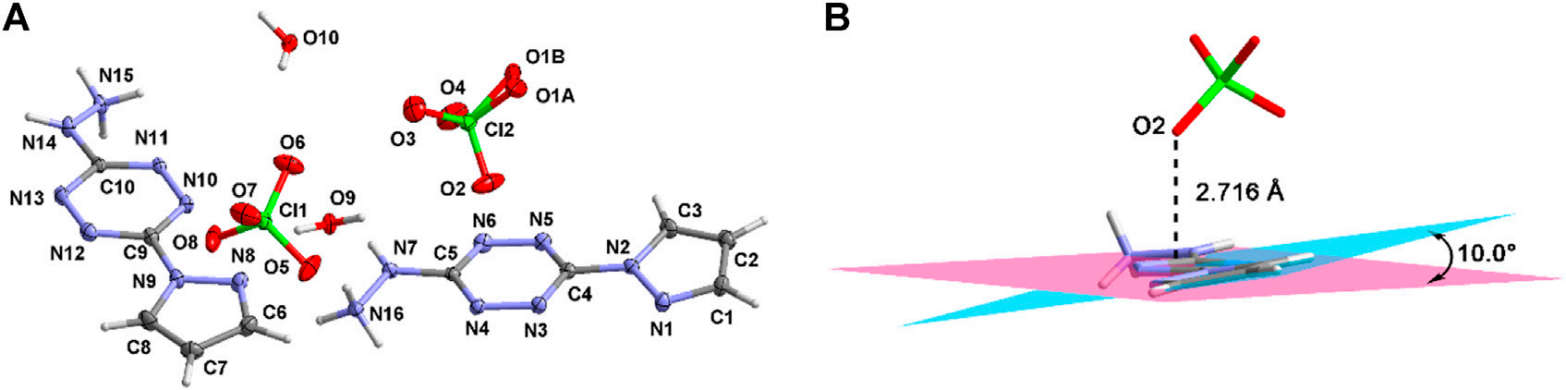
The asymmetric structural unit of **4·H**_**2**_ **O (A)** and the σ–π interactions within **4·H**_**2**_ **O (B)**.

Compound **4·H**_**2**_ **O** crystallizes in the triclinic crystal system and the *P*-1 space group. There are two cations, two anions, and two crystalline water molecules in the asymmetric structural unit (Figure 2A). The thermal vibration of the oxygen atom during the test causes the disorder of O1. The pyrazole moiety forms a dihedral angle of about 10.0° with the tetrazine ring (Figure 2B). This arises from the hydrogen-bonding interactions between the pyrazole moiety and the perchlorate ions. The bond lengths are summarized in Supplementary Table S2. As can be seen, nearly all the bond lengths of N-N, N-C, and C-C are located in the range of 1.3–1.4 Å, which reveals the existence of large conjugate structure in **4·H**_**2**_ **O**. Beyond that, the distance between the perchlorate anions and the tetrazine ring is 2.716 Å. This shows that there are σ–π interactions within the cations and the anions.

**FIGURE 3:**
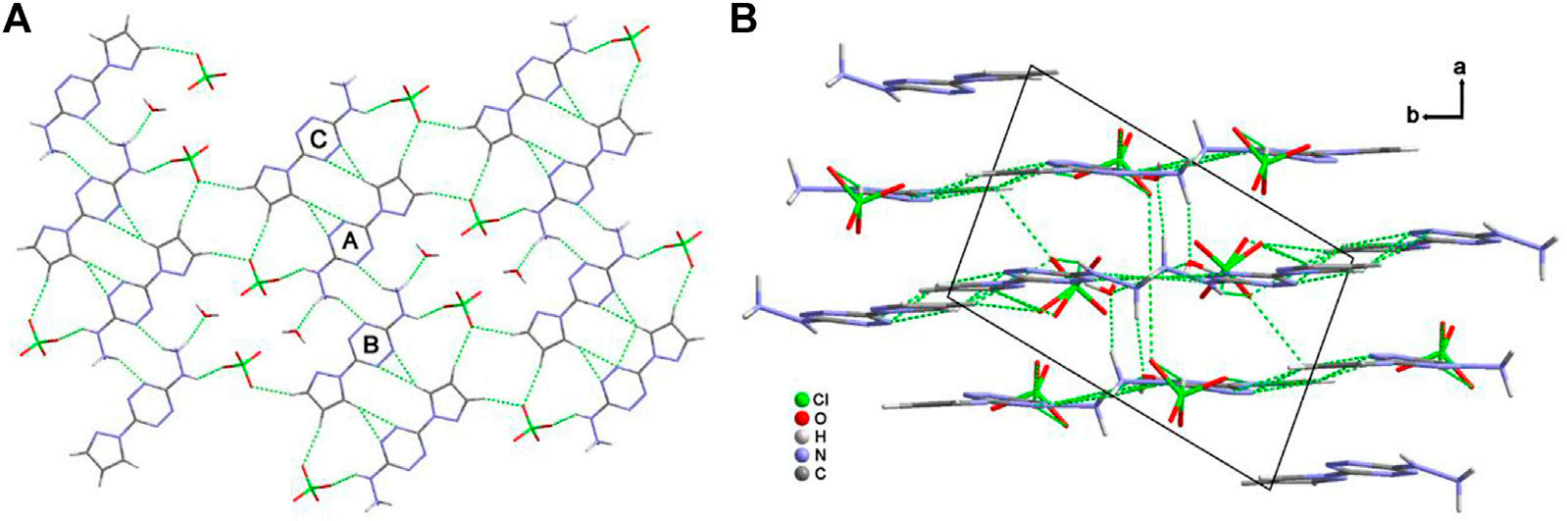
The two-dimensional hydrogen-bonding network along the *b* axis of **4·H**_**2**_ **O (A)** and the crystal packing along *c* axis of **4·H**_**2**_ **O (B)**.

Figure 3A shows the two-dimensional (2D) network of **4·H**_**2**_ **O**. The cations, anions, and crystalline water molecules connect each other through the hydrogen-bonding interactions. Among them, every two cations link each other through the “heat-to-head” (A-B) and “back-to-back” (A-C) connecting modes. Figure 3B exhibits the three-dimensional (3D) structure of **4·H**_**2**_ **O**. The σ–π interactions, π–π planar interactions, and interlayer hydrogen bonds contribute to the formation of the crystal packing of **4·H**_**2**_ **O**.

**FIGURE 4:**
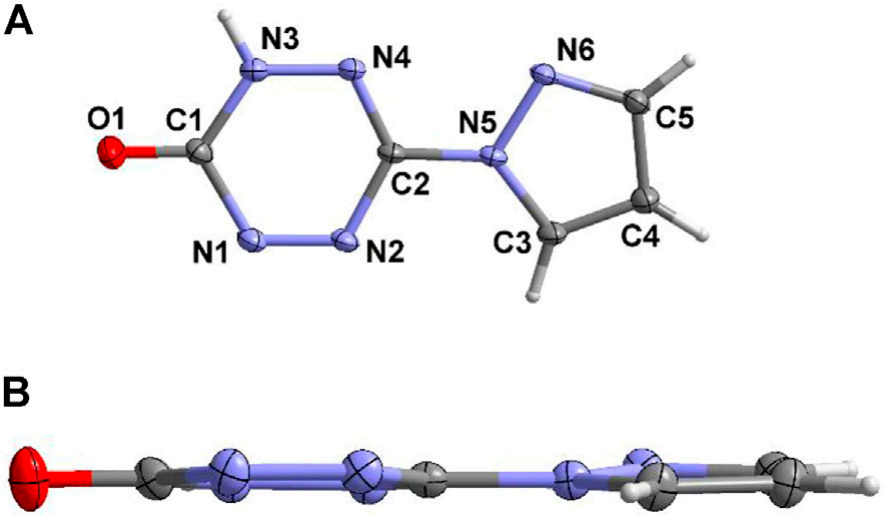
The asymmetric structural unit of **6 (A)** and its molecular planarity **(B)**.

Compound **6** crystallizes in a monoclinic crystal system and *P*2_1_/*n* space group. Due to the instability of the enol structure, the final structure of compound **5** turns out to be the ketone structure **6**, and there is only one complete molecule in the asymmetric structural unit (Figure 4A). The bond lengths of compound **6** are listed in Supplementary Table S3. The distribution of the bond lengths of N-N, N-C, and C-C are similar to that of compound **4·H**_**2**_ **O**, which lies between 1.3 and 1.4 Å. This also reveals the existence of large conjugation effect in compound **6**. Furthermore, benefiting from the large conjugate system of compound **6**, it shows good molecular planarity (Figure 4B).

**FIGURE 5:**
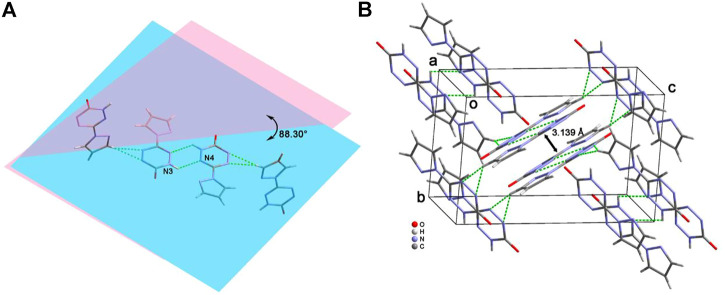
The “head-to-head” connecting mode of **6 (A)** and its packing structure **(B)**.

Due to the keto-enol tautomerism of compound **6**, each of the two molecules link with each other through the “head-to-head” connecting mode by the hydrogen-bonding rings (Figure 5A). In addition, these dimers further form a dihedral angle of about 88.3°, and they connect each other by the hydrogen bonds between pyrazole moiety and the tetrazine ring. Figure 5B exhibit the 3D structure of compound **6**. The aforementioned dimers stack with each other by π···π interactions and hydrogen bonds to construct an interlaced layered packing structure. The distance between the layers is 3.139 Å.

### Thermal behaviors analyses

The thermal decomposition behaviors of all the compounds were studied by differential scanning calorimetry (DSC) at the heating rate of 10°C·min^−1^. The DSC curves are drawn in Supplementary Figures S1–S7. In addition, the thermal decomposition parameters are listed in Table 2.

Compound **2** decomposes at 155°C with an intense exothermic process. The peak temperature (*T*_p_) of this decomposition process is 161°C, and no melting or phase transition endothermic peak is observed. For compound **3**, the thermal stability is enhanced after the formation of energetic salt. The hydrogen bonds between the anions and the cations in **3** can improve the decomposition temperature (*T*_d_). In addition, the *T*_p_ of **3** is located at 194°C, and the exothermic process is broader than that of **2**, which again demonstrates that the thermal decomposition process is more gentle for compound **3**. The *T*_d_ of the energetic salt **4** is 178°C, and it possesses multiple exothermic decomposition processes. An endothermic peak that denotes the desolvation process was observed at 117°C. The higher *T*_d_ reveals the better thermal stability of salt **4** compared with that of the neutral compound **2**. The DSC curves also show that the perchlorate salt 4 exhibits a much more complex decomposition process than the nitrate salt 3. The abundant π–π planar interactions and the interlayer hydrogen bonds in compound 4 may contribute much to this phenomenon.

When the hydrazine group is replaced by carbonyl, the thermal stability is improved. Compound **6** decomposes at 179°C, and no melting or phase transition process is observed. The ammonium salt **7** shows different thermal behavior with compounds **2**∼**5**. It decomposes at 188°C, and a melting endothermic peak appears, which is similar to the decomposition behavior of HMX. However, the hydroxylammonium (**8**) and the hydrazinium (**9**) salt show lower *T*_d_ compared with the precursor, which can also be seen in many other research works. Compound **8** decomposes at 127°C, and the decomposition residues volatilize at 305°C. For hydrazinium salt **9**, it decomposes at 133°C, and the first exothermic peak is a rapid decomposition process. After that, a second decomposition process is observed at 211°C.

### Thermodynamic parameters

Thermodynamic parameters, such as the activation energy (*E*), pre-exponential factor (*A*), activation of Gibbs free energy (Δ*G* ^≠^), activation enthalpy (Δ*H* ^≠^), and activation entropy (Δ*S* ^≠^), are important properties for energetic materials. For those compounds that possess a stable baseline and a complete single exothermic peak for the first decomposition process, *E* and *A* can be calculated using the Kissinger (Equation 1) and Ozawa (Equation 2) methods, while Δ*G* ^≠^, Δ*H* ^≠^, and Δ*S* ^≠^ can be obtained by Equations 3–5, where *β* and *R* are the heating rate and molar gas constant, respectively. *T*_e0/p0_ are temperatures when *β* approaches zero, and *h* and *k*_B_ are Planck and Boltzmann constants (Liu et al., 2017), respectively.

$$\ln\left(\frac{\beta }{T_{p}^{2}}\right)=\ln\frac{A_{k}R}{E_{k}}-\frac{E_{k}}{RT_{p}},$$ (1)

$$\log\beta +\frac{0.4567E}{RT}=C,$$ (2)

$$∆G^{\neq }=E_{k}-RT_{p0}\ln\frac{A_{k}h}{k_{B}T_{p0}},$$ (3)

$$∆H^{\neq }=E_{k}-RT_{p0},$$ (4)

$$∆S^{\neq }=\frac{∆H^{\neq }-∆G^{\neq }}{T_{p0}}.$$ (5)

The calculated results are summarized in Table 1 and Supplementary Table S4. *T*_e_ and *T*_p_ used for calculating the parameters in Table 1 at different heating rates are listed in Supplementary Table S5. As can be seen, when the hydrazine group is replaced by carbonyl, the values of Δ*G* ^≠^, Δ*H* ^≠^, and Δ*S* ^≠^ are all improved, demonstrating that the thermal safety of compound **6** is better than that of **2**. As for the energetic salts of **6**, the order of thermal safety is **7** > **9** > **8**, which is consistent with their thermal behaviors.

**TABLE 1:** The kinetic parameters of compounds **2** and **6**–**9**.

Compounds	*E*_op_ (kJ·mol^−1^)	*r*_op_	*E*_k_ (kJ·mol^−1^)	log*A*_k_ (s^−1^)	*r*_k_	*E*_oe_ (kJ·mol^−1^)	*r*_oe_
**2**	163.4 ± 6.3	0.997	164.6 ± 6.3	18.1 ± 0.7	0.997	172.6 ± 1.4	0.992
**6**	232.6 ± 3.0	0.998	237.0 ± 4.0	25.5 ± 0.4	0.998	226.3 ± 0.6	0.996
**7**	229.8 ± 5.1	0.996	233.9 ± 5.3	24.7 ± 0.7	0.995	230.5 ± 1.9	0.994
**8**	149.5 ± 6.4	0.986	150.5 ± 3.7	17.7 ± 0.5	0.985	156.4 ± 3.0	0.995
**9**	210.9 ± 6.2	0.990	215.0 ± 6.5	25.9 ± 0.8	0.990	206.7 ± 8.3	0.995

Note: *E*, activation energy; *r*, linear correlation coefficient; *A*, exponential factor. The subscript with op indicates parameters calculated by the Ozawa method using peak temperature; the subscript k indicates parameters calculated by the Kissinger method using peak temperature; and the subscript oe indicates parameters calculated by the Ozawa method using extrapolated temperature.

### Energetic property and sensitivity

The densities of **2**–**4** and **6**–**9** were determined in order to obtain their energetic properties. The results are listed in Table 2. The density of compound **4** is the highest because of the higher molecular weight of ClO_4_^−^. The density of **6** is greater than that of 5, which may benefit from its stronger intermolecular hydrogen bonds. Except for compounds **2** and **3**, the densities of other compounds are all higher than that of TNT.

**TABLE 2:** Physical properties of obtained **2**–**4** and **6**–**9**.

Compound	*T*_d_ ^a^ (°C)	*ρ* ^b^ (g·cm^−3^)	Δ*H*_f_ ^c^ (kJ·mol^−1^/kJ·g^−1^)	*D* ^d^ (m·s^−1^)	*P* ^e^ (GPa)	IS^f^ (J)
**2**	155	1.60	702.6/3.94	7,615	19.6	30
**3**	175	1.63	674.9/2.80	7,929	23.9	14
**4**	178	1.73	715.4/2.57	8,003	26.5	6
**6**	179	1.69	403.2/2.46	6,966	16.8	30
**7**	188	1.67	378.5/2.09	7,706	20.4	36
**8**	127	1.69	431.5/2.19	7,893	22.5	32
**9**	133	1.68	532.2/2.71	8,232	23.6	32
**TNT**	295.0	1.65	−59.4/−0.26	7,303	21.3	15
**RDX**	204	1.80	81/0.36	8,795	34.9	7.2
**TATB**	350	1.86	−154.2/−0.6	8,114	29.1	—
**HNS**	311	1.73	—	7,170	21.8	—

a Decomposition temperature (onset, 10°C min^−1^).

b Density measured using a gas pycnometer (25°C).

c Heat of formation.

d Detonation velocity (calculated using Explo5 v6.04).

e Detonation pressure (calculated using Explo5 v6.04).

f Impact sensitivity.

The heats of formation (Δ*H*_f_) of all the compounds were obtained theoretically based on isodesmic reactions. As can be seen in Table 2, all the compounds possess positive Δ*H*_f_ that is much higher than that of TNT. In addition, compounds **2**–**4** show greater Δ*H*_f_ than **6**–**9**, which is because the Δ*H*_f_ of the hydrazine group is higher than that of carbonyl.

Based on the obtained Δ*H*_f_ and densities, the energetic performances, including detonation velocity (*D*) and detonation pressure (*P*), are calculated using Explo5 v6.04. (Suceska, 2017). As can be seen in Table 2, compound **6** exhibits the lowest detonation performances (*D* = 6,966 m s^−1^ and *p* = 16.8 GPa), while the *D* of other compounds are higher than 7,000 m s^−1^ and greater than that of TNT. The *P* of compounds **2** and **6** is 19.6 and 16.8 GPa, while the *P*s of other explosives are compared with the *P* of TNT. The hydrazinium salt **9** has the best detonation properties (*D* = 8,232 m s^−1^ and *p* = 23.6 GPa) among all the synthesized compounds, which are better than those of TNT and HNS, compared to TATB, but lower than RDX. Furthermore, the energetic performances of all the energetic salts (**3**, **4**, and **7**–**9**) are better than the neutral compounds (**2** and **6**), revealing that the synthesis of energetic salts is an effective way to improve the energy of explosives.

The impact sensitivities (IS) of all the explosives were determined on the basis of the UN Recommendations on the Transport of Dangerous Goods, Manual of Tests, and Criteria (Manual of Tests and Criteria, 2009). The perchlorate salt **4** is sensitive to the external impact, which is consistent with the finding of previous work. The IS of nitrate salt **3** is 14J, which is comparable to TNT. Except for compounds **3** and **4**, all the other explosives are insensitive to impact, and the IS are all above 30 J, which may be used as insensitive explosives.

**FIGURE 6:**
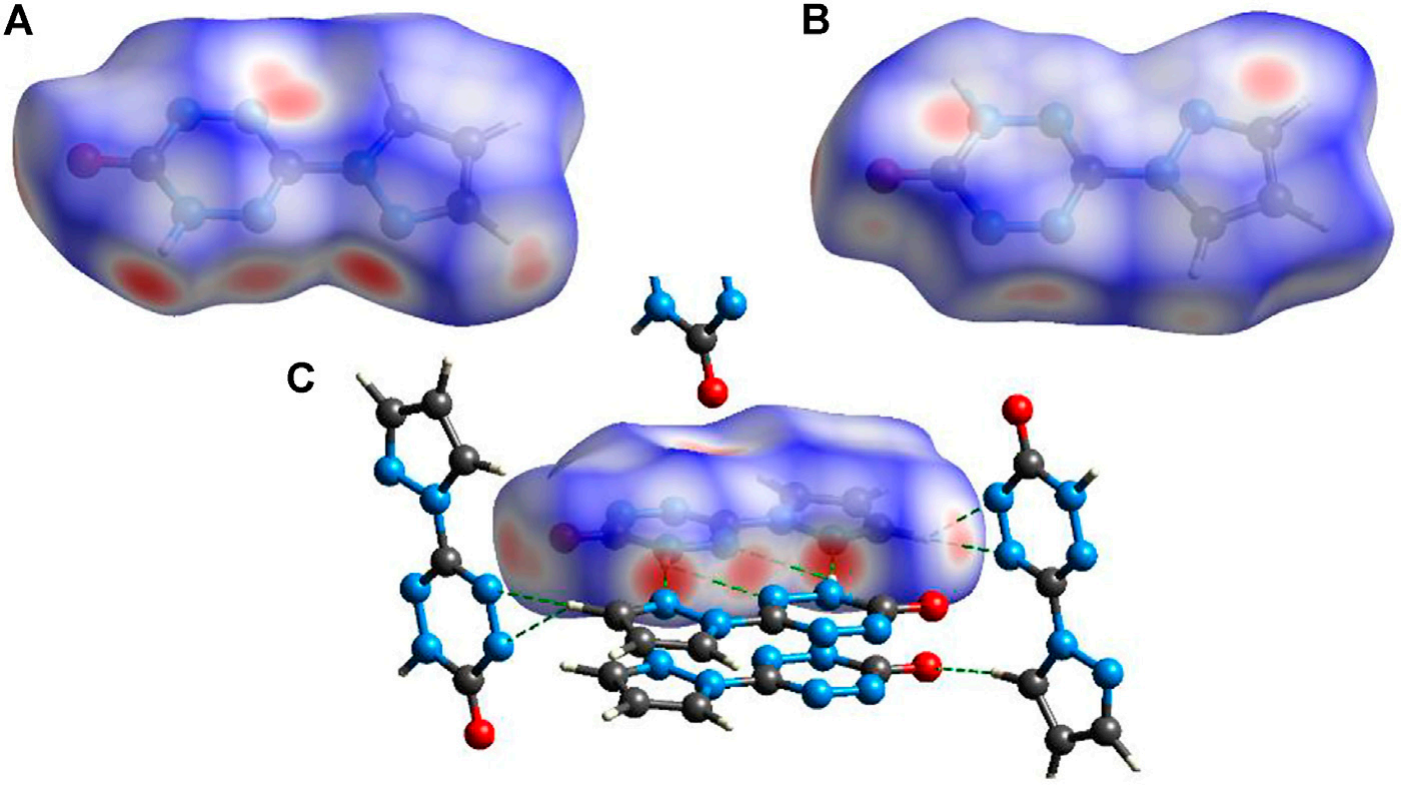
Hirshfeld surfaces viewed from the top **(A)** and bottom **(B)**. **(C)** Intermolecular interactions around compound **6**.

**FIGURE 7:**
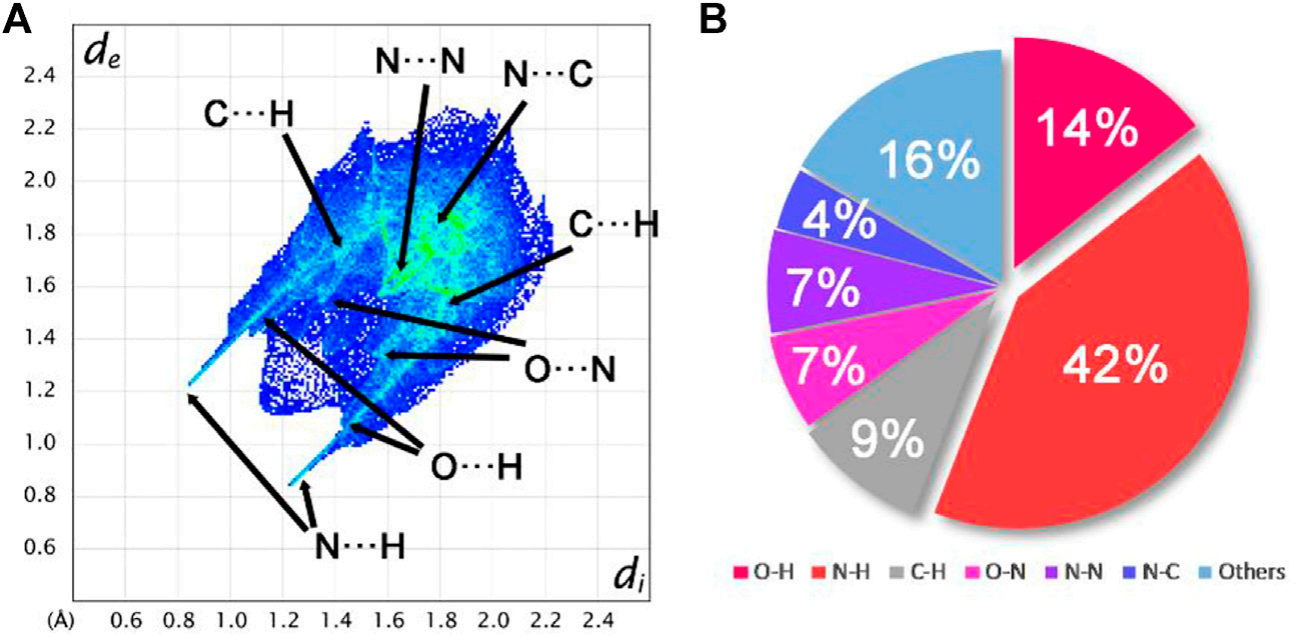
**(A)** 2D fingerprint plot for compound **6**. **(B)** Populations of the molecular contacts for compound **6**.

To explain the mechanical stability of **2** and **6**–**9**, noncovalent interaction analyses were carried out based on the crystal structure of **6**. Figure 6 exhibits the Hirshfeld surfaces analysis of **6**, while Figure 7 shows its 2D fingerprint plot and the populations of the molecular contacts. As can be seen in Figure 6, the Hirshfeld surface of **6** presents a flat structure. In addition, the red dots that denote hydrogen bonds are mostly distributed around the surface. For energetic materials, the flatter the surface is and the more red areas located around the surface, the higher the stability of explosives is. Furthermore, the 2D fingerprint plot of **6** clearly shows a pair of spikes that denotes the strong N–H interactions, and it occupies up to 42% of the molecular contacts. This strong and high proportion of hydrogen bonds contributes to buffering external mechanical stimuli, which plays an important role in the low IS of compound **6**. This may also be the reason for the mechanical stability of **2** and **7**–**9**.

## Conclusion

Two asymmetrically substituted tetrazines were prepared. Their five energetic salts were first synthesized and fully characterized *via* IR spectroscopy, NMR spectra, elemental analysis, and differential scanning calorimetry (DSC). Single-crystals of **4·H**_**2**_ **O** and **6** were successfully obtained, and their structures were further studied by single-crystal X-ray diﬀraction. The homogenized bond lengths disclose the large conjugate system existing in these two compounds. The thermal decomposition behaviors of all the compounds were studied, and their decomposition temperatures range from 127 to 188°C. The ammonium salt **7** shows the best thermal stability, and it melts before decomposition, while other compounds decompose directly. The thermodynamic parameters of **2** and **6**–**9** were calculated, and the order of thermal safety is **7** > **6** > **2** > **9** > **8**. All the compounds exhibit positive Δ*H*_f_ that is much higher than that of TNT. Compounds **2**–**4** and **7**–**9** show higher detonation velocities than TNT, and the hydrazinium salt **9** has the best detonation properties (*D* = 8,232 m s^−1^ and *p* = 23.6 GPa). Compound **4** is sensitive to impact, while **3** shows impact sensitivity comparable to TNT. In addition, other compounds are insensitive to impact; noncovalent interaction analysis based on **6** reveals that the strong and high proportion of hydrogen bonds could contribute to their low-impact sensitivity, and these compounds may be applied in insensitive explosives.

## Data Availability

The original contributions presented in the study are included in the article/Supplementary Material; further inquiries can be directed to the corresponding author.
