# Supplementary material for: Synthesis, thermal behaviors, and energetic properties of asymmetrically substituted tetrazine-based energetic materials
Source: Front Chem. 2022 Oct 3;10:978003. doi: 10.3389/fchem.2022.978003 (PMC9574065; doi:10.3389/fchem.2022.978003)
Supplement: Supplementary file 1 [file DataSheet1.docx]

Synthesis, thermal behaviors and energetic properties of asymmetrically substituted tetrazine-based energetic materials

*Shenghui Wang^a,1^, Xiang Chen ^a,1^, Yuankai Chen^a^, Hai Nan^b^, Yuanyuan Li^b^, Haixia Ma^a^^[[1]](#footnote-1)^**

*a. School of Chemical Engineering / Xi'an Key Laboratory of Special Energy Materials, Northwest University,* *Xi'an 710069, Shaanxi, P. R. China.*

*b. Xi’an Modern Chemistry Research Institute, Xi’an 710065, P. R. China*

**Table of Contents**

1. Crystallographic data.

2. DSC curves.

3. Computational details.

4. NMR spectra.

5. References.

**1. Crystallographic data**

**Table S1** Crystallographic data for **4·H_2_O** and **6**

| Compound | **4·H_2_O** | **6** |
| --- | --- | --- |
| Empirical formula | C_5_H_9_ClN_8_O_5_ | C_5_H_4_N_6_O |
| Temperature/K | 296 | 296 |
| Crystal system | triclinic | monoclinic |
| Space group | *P*-1 | *P*2_1_/*n* |
| *α*/° | 89.269(3) | 90 |
| *β*/° | 87.478(4) | 98.097(2) |
| *γ*/° | 78.905(3) | 90 |
| *a*/Å | 7.5474(8) | 5.1444(3) |
| *b*/Å | 11.5014(11) | 9.2378(7) |
| *c*/Å | 13.4493(13) | 13.7906(8) |
| Volume/Å^3^ | 1144.5(2) | 648.84(7) |
| *Z* | 4 | 4 |
| *μ*/mm^‑1^ | 0.371 | 0.130 |
| *F*(000) | 608.0 | 336.0 |
| Density/g·cm^-3^ | 1.722 | 1.680 |
| 2θ range/° | 4.704 to 51.998 | 5.968 to 72.534 |
| Data/restraints/parameters | 4393/1/362 | 3111/0/109 |
| Reflections collected | 4393 | 10477 |
| Independent reflections | 4393 | 3111 |
| GOOF | 1.060 | 1.032 |
| Final *R* indexes [I>=2*σ*(*I*)] | R_1_ = 0.0812, wR_2_ = 0.2157 | R_1_ = 0.0498, wR_2_ = 0.1220 |
| Final *R* indexes [all data] | R_1_ = 0.0964, wR_2_ = 0.2247 | R_1_ = 0.0782, wR_2_ = 0.1400 |
| Recrystallization solvent | Filtrate | Filtrate |
| CCDC | 2128980 | 2128981 |

**Table S2** The bond lengths of **4·H_2_O**

| Bond | Length/Å | Bond | Length/Å | Bond | Length/Å |
| --- | --- | --- | --- | --- | --- |
| N15-N14 | 1.414(5) | C7-C6 | 1.403(8) | N4-C5 | 1.319(6) |
| N9-N8 | 1.359(6) | N14-C10 | 1.363(6) | N6-C5 | 1.353(6) |
| N9-C8 | 1.371(6) | N2-N1 | 1.361(5) | C2-C3 | 1.352(7) |
| N9-C9 | 1.385(6) | N2-C4 | 1.387(6) | Cl1-O6 | 1.427(4) |
| N10-N11 | 1.330(5) | N2-C3 | 1.366(6) | Cl1-O5 | 1.433(4) |
| N10-C9 | 1.315(6) | N5-C4 | 1.339(6) | Cl1-O8 | 1.424(4) |
| N11-C10 | 1.316(6) | N5-N6 | 1.296(6) | Cl1-O7 | 1.410(4) |
| N12-C9 | 1.350(6) | N3-C4 | 1.327(6) | Cl2-O3 | 1.423(4) |
| N8-C6 | 1.320(7) | N7-N16 | 1.415(5) | Cl2-O2 | 1.420(5) |

**Table S3** The bond lengths of **6**

| Bond | Length/Å | Bond | Length/Å | Bond | Length/Å |
| --- | --- | --- | --- | --- | --- |
| O1-C1 | 1.2096(14) | N4-C2 | 1.2934(13) | N2-C2 | 1.3770(12) |
| N5-N6 | 1.3691(11) | N6-C5 | 1.3233(15) | N1-C1 | 1.4253(15) |
| N5-C2 | 1.3896(13) | N3-C1 | 1.3645(13) | C3-C4 | 1.3642(16) |
| N5-C3 | 1.3636(14) | N2-N1 | 1.2794(13) | C5-C4 | 1.4024(16) |
| N4-N3 | 1.3383(13) |  |  |  |  |

**2. DSC curves**

**

**

**Fig. S1** The DSC curves of compound **2**

**

**

**Fig. S2** The DSC curves of compound **3**

**

**

**Fig. S3** The DSC curves of compound **4**

**

**

**Fig. S4** The DSC curves of compound **6**

**

**

**Fig. S5** The DSC curves of compound **7**

**

**

**Fig. S6** The DSC curves of compound **8**

**

**

**Fig. S7** The DSC curves of compound **9**

**Table S4** The thermal safety parameters of compound **2**, **6-9**.

| Compound | *T*_p0_ (^o^C) | *T*_e0_ (^o^C) | Δ*S*^≠^  (J·mol^-1^·K^-1^) | Δ*G*^≠^  (kJ·mol^-1^) | Δ*H*^≠^  (kJ·mol^-1^) |
| --- | --- | --- | --- | --- | --- |
| **2** | 148.3 | 143.8 | 89.4 | 123.4 | 161.1 |
| **6** | 173.0 | 170.0 | 231.7 | 129.9 | 233.3 |
| **7** | 180.5 | 177.6 | 215.3 | 132.5 | 230.1 |
| **8** | 121.6 | 112.7 | 82.9 | 114.5 | 147.3 |
| **9** | 127.1 | 120.3 | 240.5 | 115.8 | 211.6 |

**Table S5** The *T*_e_ and *T*_p_ at different heating rates.

| Compound | *β* (^o^C·min^-1^) | *T*_e_ (^o^C) | *T*_p_ (^o^C) |
| --- | --- | --- | --- |
| **2** | 5 | 150.50±0.44 | 154.75±0.07 |
|  | 10 | 155.01±0.30 | 160.74±0.03 |
|  | 15 | 159.85±0.33 | 164.02±0.23 |
|  | 20 | 161.68±0.17 | 167.63±0.62 |
| **6** | 5 | 174.83±0.02 | 178.16±0.06 |
|  | 10 | 179.14±0.19 | 182.54±0.18 |
|  | 15 | 182.13±0.76 | 185.57±0.18 |
|  | 20 | 184.85±0.44 | 188.01±0.22 |
| **7** | 5 | 182.00±0.15 | 186.32±0.15 |
|  | 10 | 186.20±0.05 | 190.54±0.22 |
|  | 15 | 189.14±0.21 | 194.48±0.12 |
|  | 20 | 192.18±0.04 | 196.32±0.25 |
| **8** | 5 | 121.62±0.06 | 126.28±0.02 |
|  | 7.5 | 124.45±0.38 | 129.37±0.12 |
|  | 10 | 127.43±0.40 | 131.11±0.42 |
|  | 12.5 | 128.67±0.10 | 134.32±0.04 |
| **9** | 5 | 128.81±0.27 | 131.10±0.09 |
|  | 7.5 | 131.74±0.42 | 132.85±0.51 |
|  | 10 | 133.43±0.34 | 134.98±0.93 |
|  | 12.5 | 134.45±0.06 | 136.67±0.06 |

**3. Computational details**

The theoretical calculations were performed on Gaussian 16 program (Revision C.01). Geometric optimization and frequency analyses were worked at B3LYP/6-31+G** level. Moreover, the single point energies of all the explosives were performed at MP2/6-311++G** level.^1^ Atomization energies were calculated by G2 ab initio method on the basis of atomization reaction and NIST WebBook.^2,3^ All of the optimized structures were confirmed to be true local energy minima without imaginary frequencies. The heat of formation (HOF) of all the compounds were obtained by isodesmic reactions (Scheme S1). The final results of the gas-phase species were list in Table S6.

**Scheme S1** The isodesmic reactions of obtained compounds.

**Table S6** Calculated HOF of the gas-phase species

| Compound | $\Delta H_{f}^{^{\circ}}$ (kJ·mol^-1^) | Compound | $\Delta H_{f}^{^{\circ}}$ (kJ·mol^-1^) |
| --- | --- | --- | --- |
| **1** | 783.12 | **2** | 478.70 |
| **3** | 177.40 | **4** | 1466.09 |
| **5** | 403.21 | **6** | 268.60 |
| **7** | 276.26 | **8** | 101.29 |

The solid-phase HOF of the neutral compounds is calculated by subtracting the heat of sublimation ($\Delta H_{\mathrm{sub}}$) from the gas-phase HOF based on Trouton’s rule by using equation 1, where *T* denotes either the melting point or the decomposition temperature when no melting occurs before the decomposition.^4^

$\Delta H_{f}\left( s \right)=\Delta H_{f}\left( g \right)-\Delta H_{\mathrm{sub}}=\Delta H_{f}\left( g \right)-188\left[ J\cdot mol\cdot K^{-1} \right] \times T$ (1)

The solid-phase HOF of the energetic salts is calculated on the basis of Born-Haber energy cycle (Scheme S2). The calculation equation is shown in equation 2.


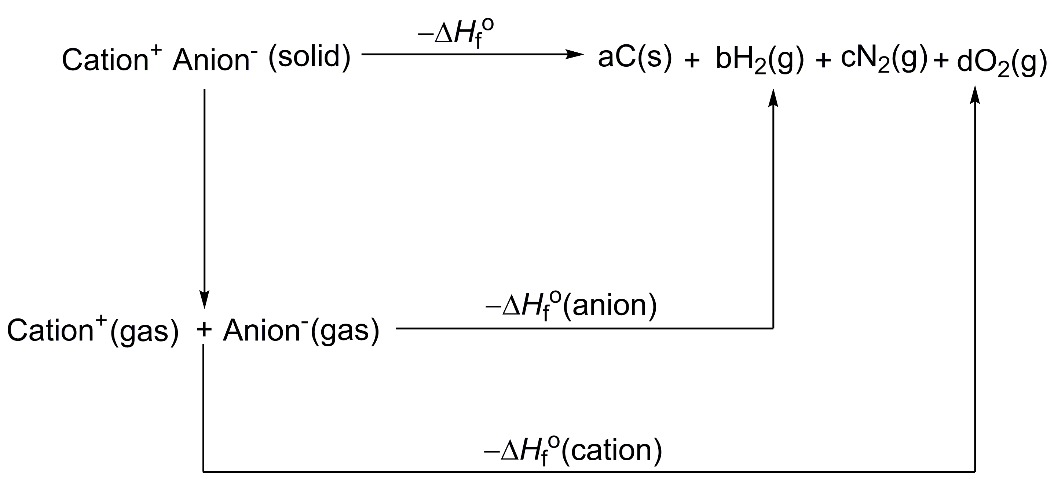


**Scheme S2** Born-Haber energy cycle for the formation of energetic salts.

$\Delta H_{f}^{o}\left( salt,298 K \right)=\Delta H_{f}^{o}\left( cation,298 K \right)+\Delta H_{f}^{o}\left( anion,298 K \right)-\Delta H_{L}$ (2)

In the above equation, $\Delta H_{L}$denotes the lattice energy of the ionic salts which can be obtained by using equation 3 that suggested by Jenkins, et al.^5^

$\Delta H_{L}=U_{\mathrm{pot}}+[p\left( \frac{n_{M}}{2}-2 \right)+q(\frac{n_{X}}{2}-2)]RT$ (3)

The values of $n_{M}$ and $n_{X}$ depend on the nature of the ions ( $M^{q+}$and $X^{P-}$), which equal to 3 for monatomic ions, 5 for linear polyatomic ions, and 6 for nonlinear polyatomic ions. $U_{\mathrm{pot}}$ is the lattice potential energy that can be obtained by using equation 4.

$U_{\mathrm{pot}}\left( \mathrm{kJ}\cdot\mathrm{mol}^{-1} \right)=\gamma{(\rho_{m}/M_{m})}^{1/3}+\delta$ (4)

In the above equation, $\rho_{m}$ is the density of the energetic salt, $M_{m}$ denotes the chemical formula mass of the salt. The values of $\gamma(\mathrm{kJ}\cdot\mathrm{mol}^{-1}\cdot\mathrm{cm})$ and $\delta(\mathrm{kJ}\cdot\mathrm{mol}^{-1})$ can be assigned according to the literature.^5^

**4. NMR spectra**


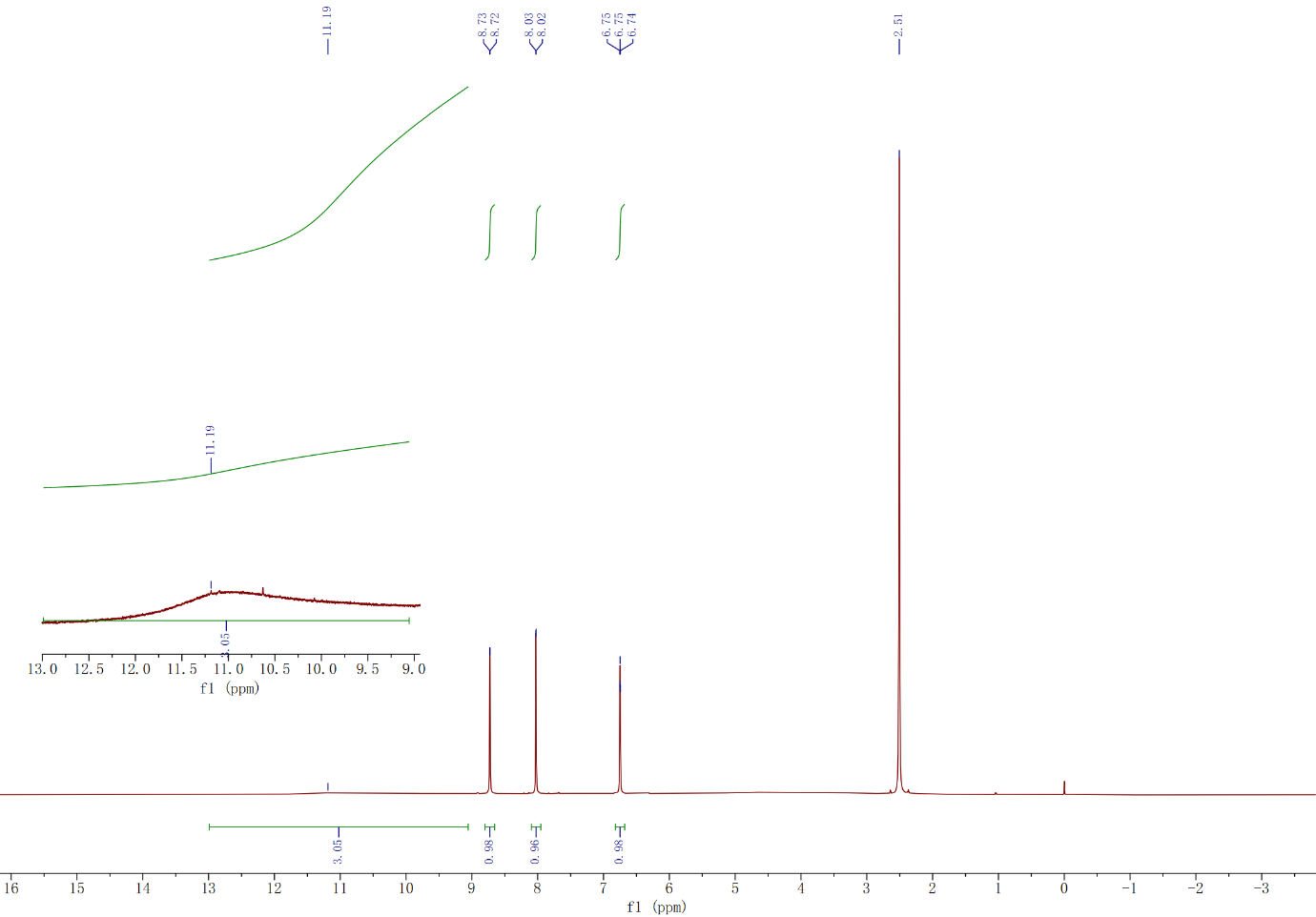


**Fig. S8** ^1^H NMR spectrum of **3**.


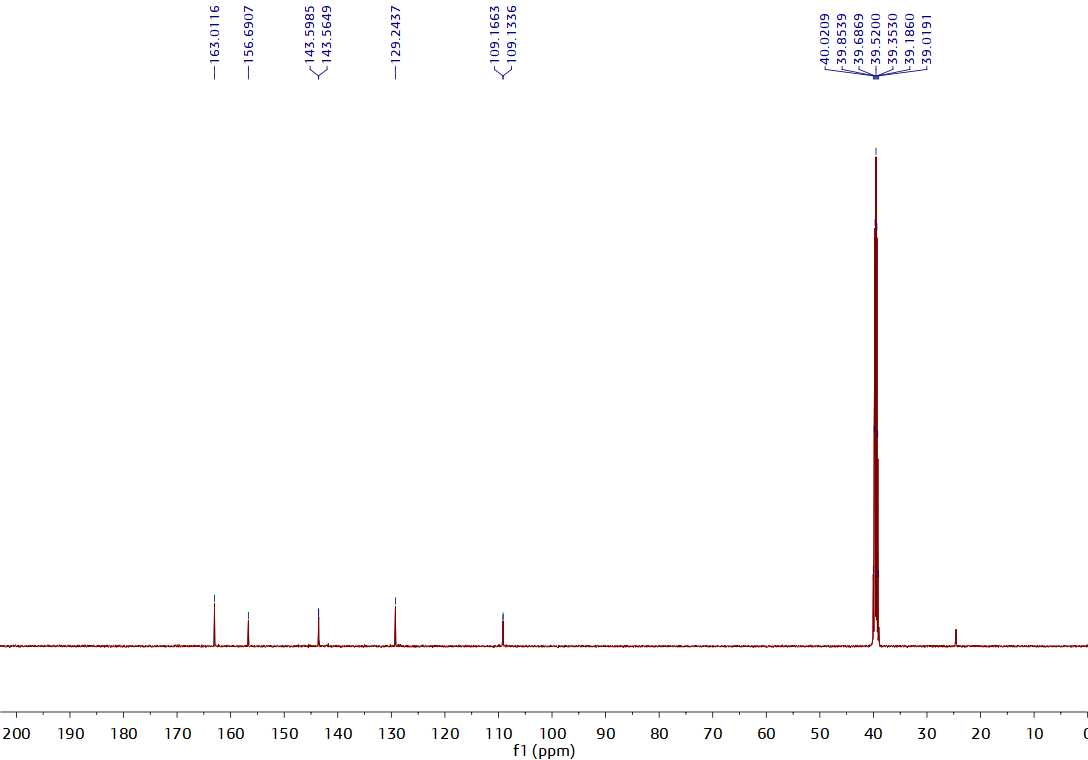


**Fig. S9** ^13^C NMR spectrum of **3**.


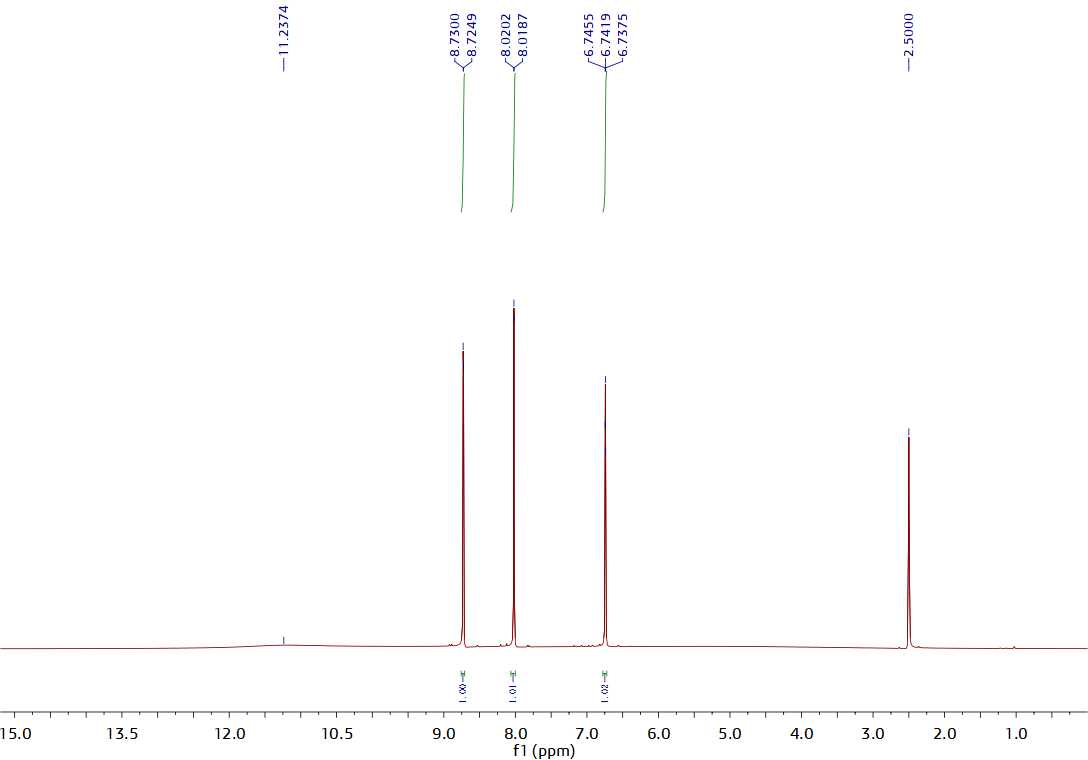


**Fig. S10** ^1^H NMR spectrum of **4**.


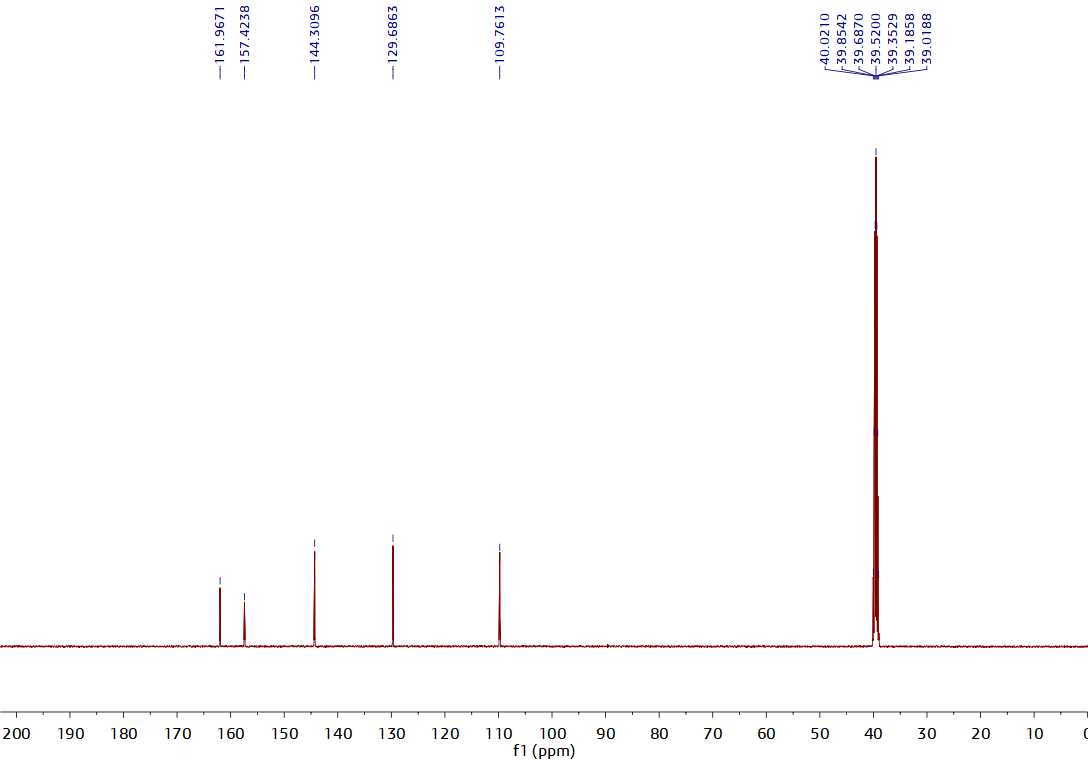


**Fig. S11** ^13^C NMR spectrum of **4**.


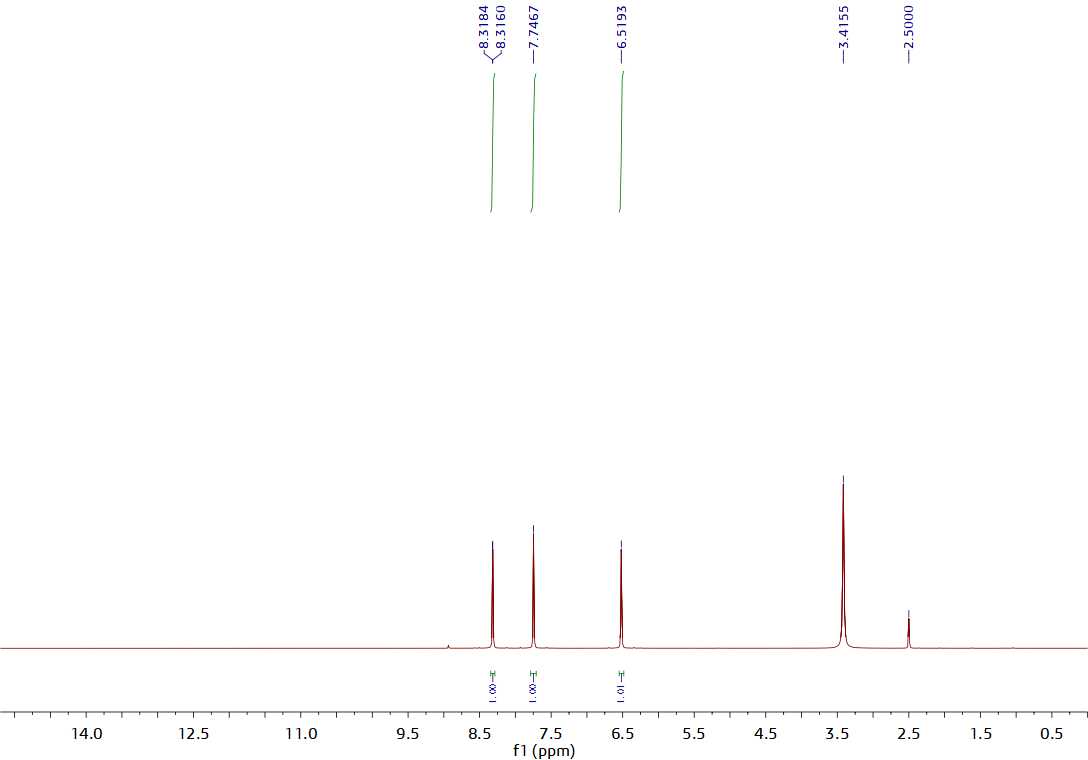


**Fig. S12** ^1^H NMR spectrum of **6**.


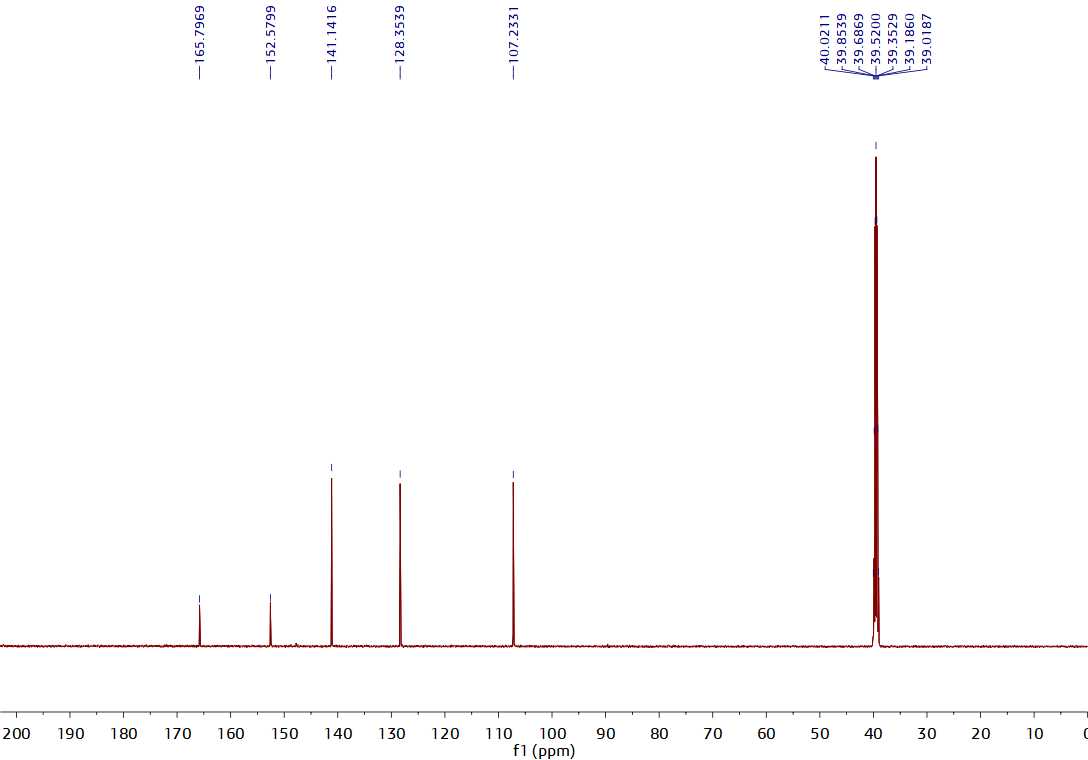


**Fig. S13** ^13^C NMR spectrum of **6**.


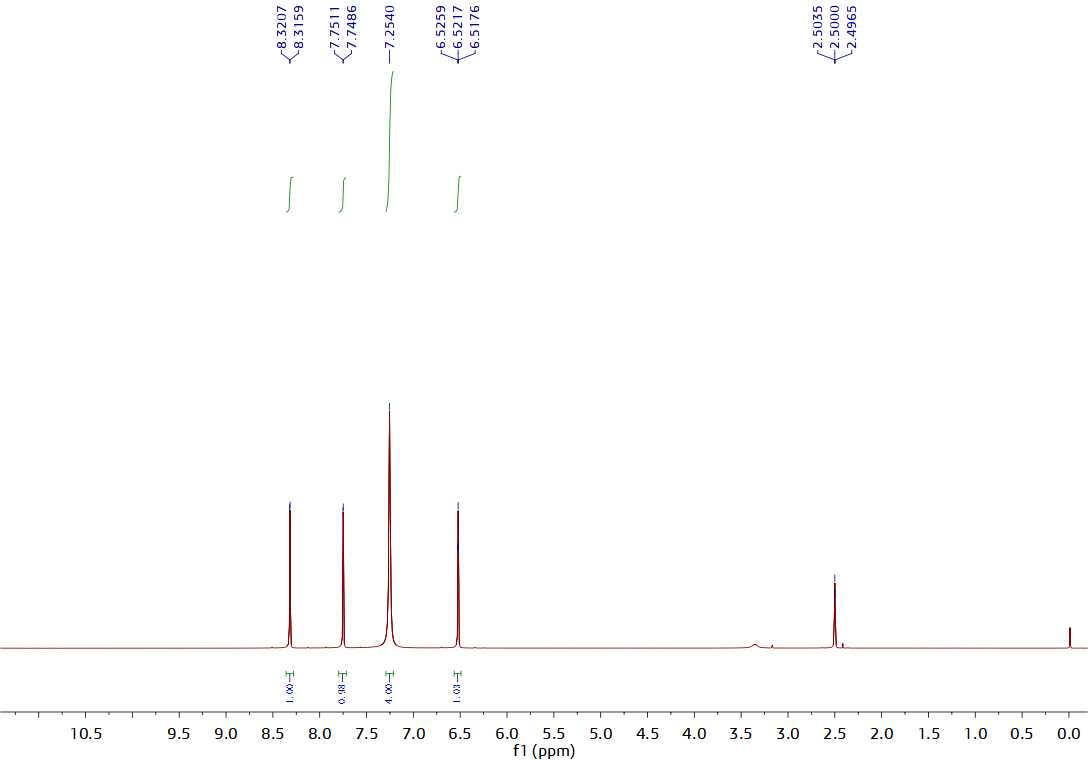


**Fig. S14** ^1^H NMR spectrum of **7**.


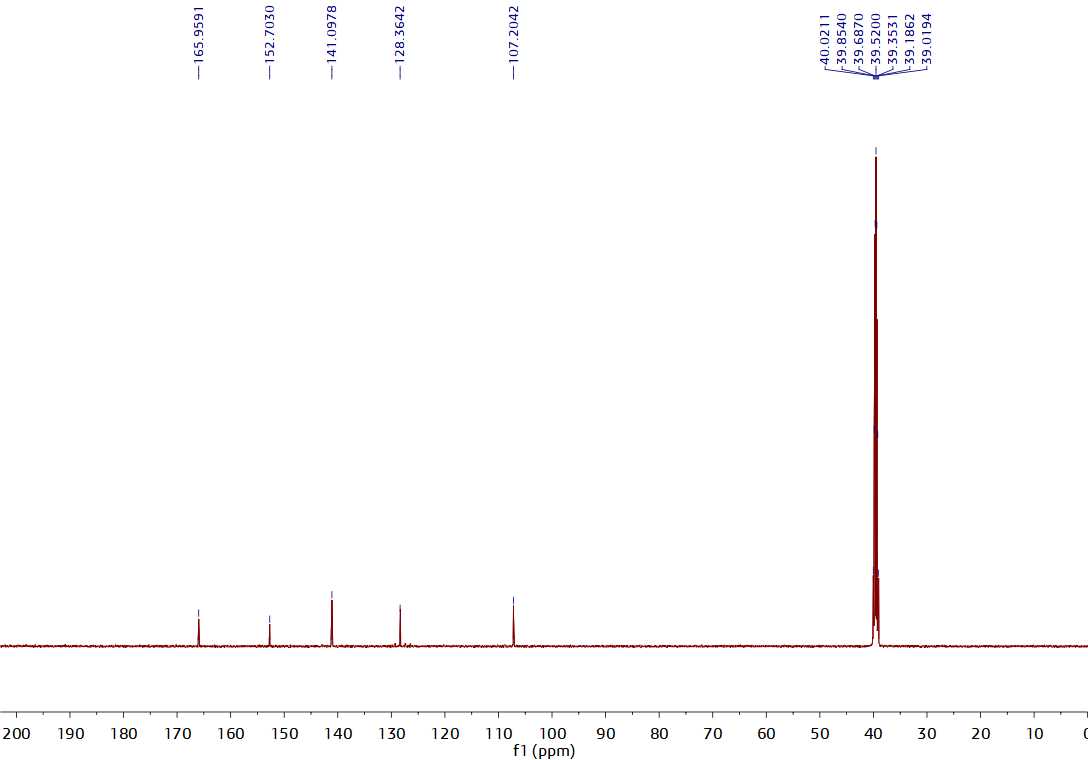


**Fig. S15** ^13^C NMR spectrum of **7**.


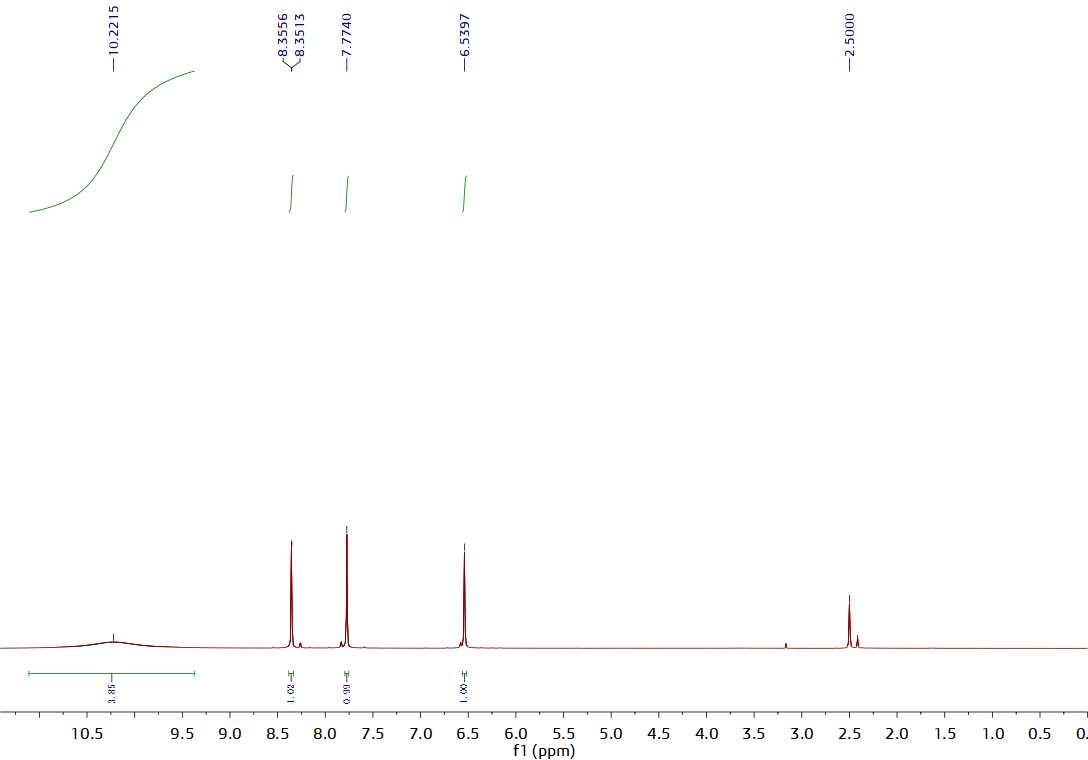


**Fig. S16** ^1^H NMR spectrum of **8**.


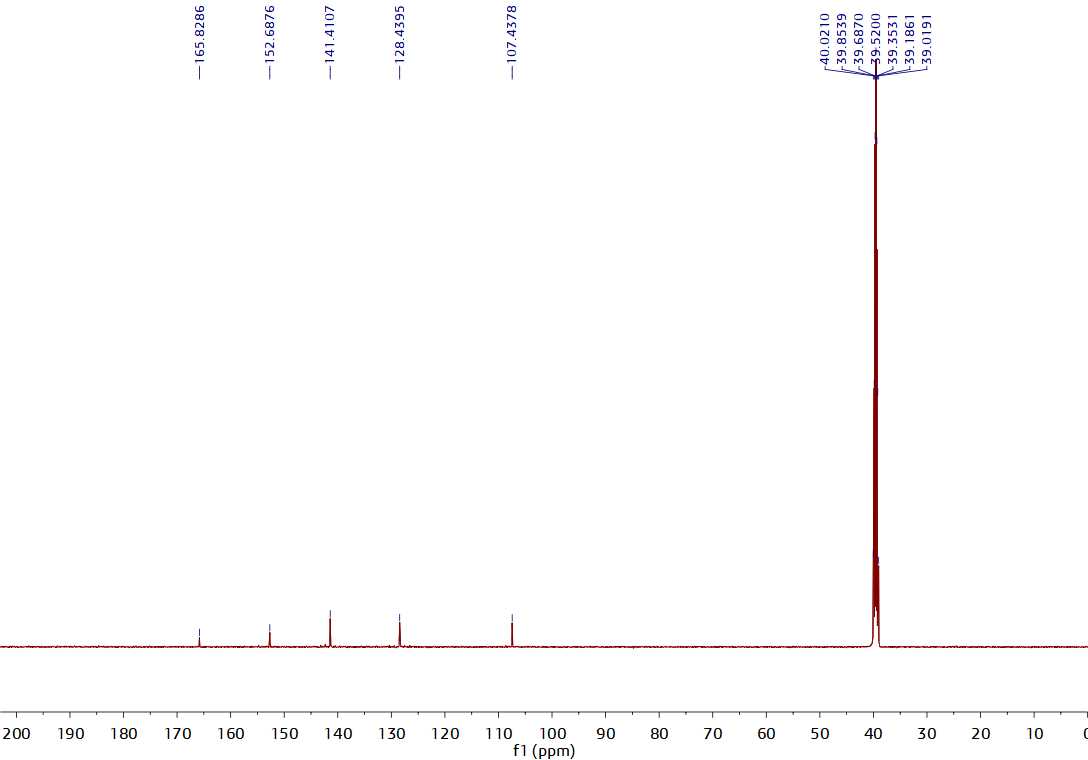


**Fig. S17** ^13^C NMR spectrum of **8**.


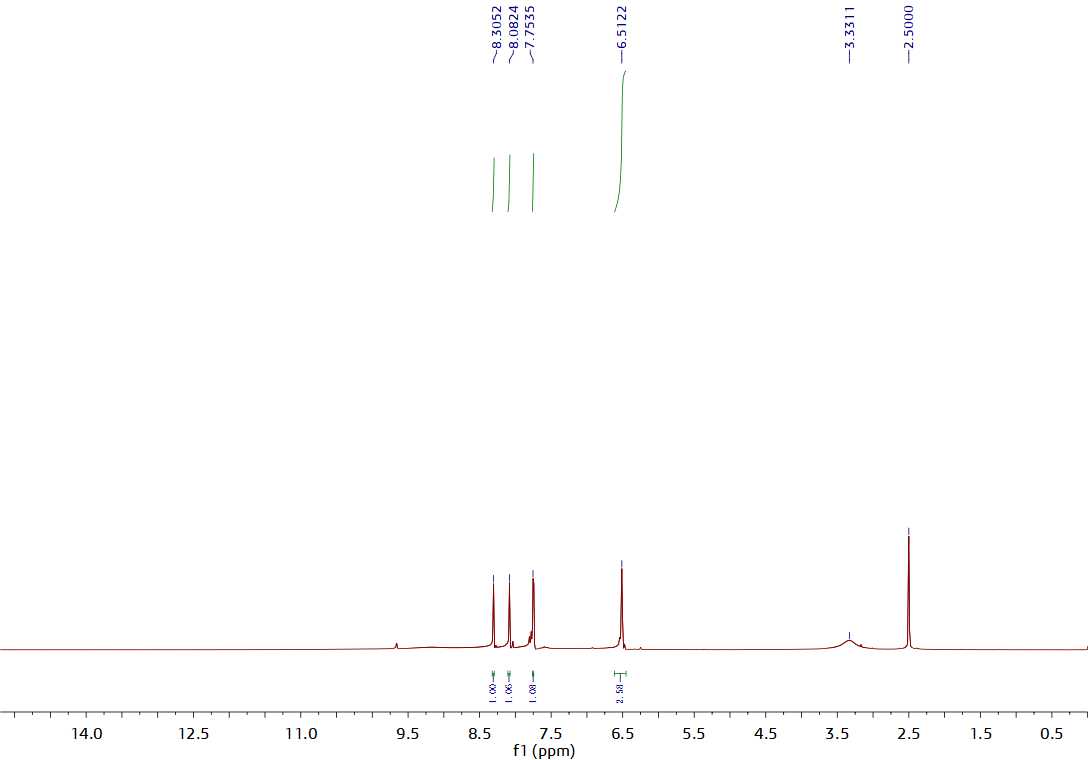


**Fig. S18** ^1^H NMR spectrum of **9**.


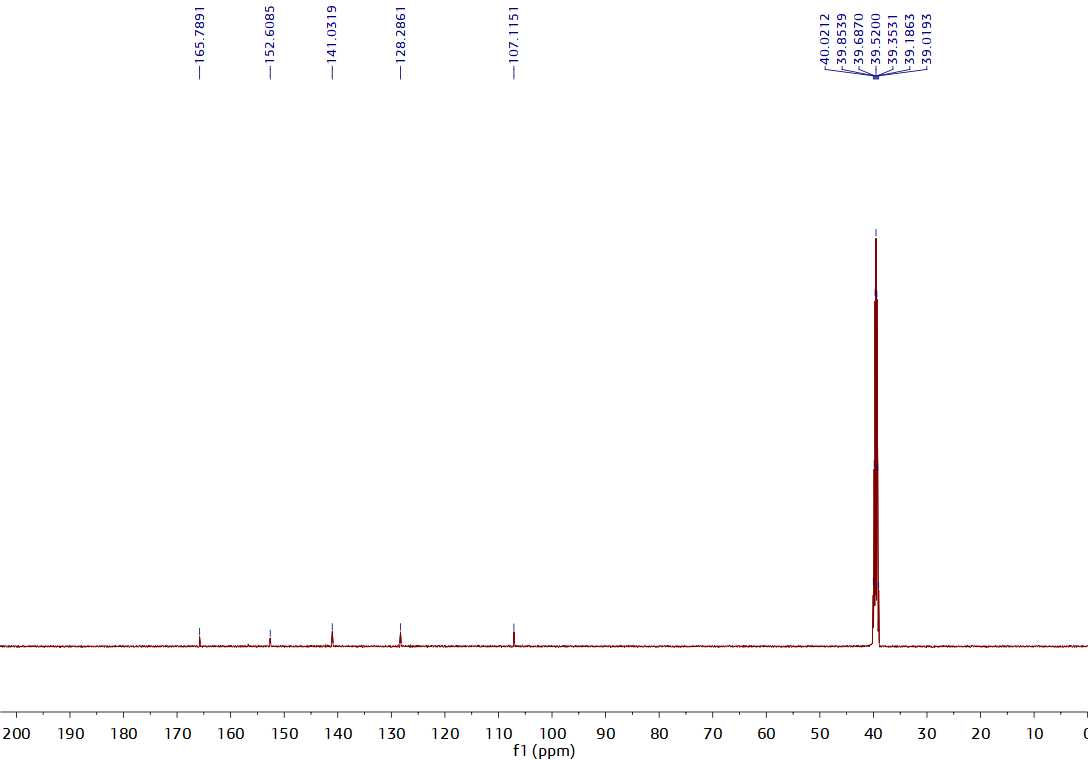


**Fig. S19** ^13^C NMR spectrum of **9**.

**5. References.**

1. R. G. Parr and W. Yang, *Density Functional Theory of Atoms and Molecules*, Oxford University Press: Oxford, U.K., 1989.
2. L. A. Curtiss, K. Raghavachari, G. W. Trucks and J. A. Pople, *J. Chem. Phys.*, 1991, **94**, 7221- 7230.
3. NIST Chemistry WebBook, <https://webbook.nist.gov/chemistry/>.
4. M. S. Westwell, M. S. Searle, D. J. Wales and D. H. Wiliams, *J. Am. Chem. Soc.*, 1995, **117**, 5013.
5. H. D. B. Jenkins, D. Tudela and L. Glasser, *Inorg. Chem.*, 2002, **41**, 2364-2367.

1. * Corresponding author. Email address: [mahx@nwu.edu.cn](mailto:mahx@nwu.edu.cn).

   1. These authors contributed equally to this work. [↑](#footnote-ref-1)
